# Child Gender and Married Women’s Overwork: Evidence from Rural–Urban Migrants in China

**DOI:** 10.3390/healthcare10061126

**Published:** 2022-06-16

**Authors:** Yanjiao Song, Ruojing Wang

**Affiliations:** 1 Center for Modern Chinese City Studies, Institute of Urban Development, East China Normal University, Shanghai 200062, China; yjsong@iud.ecnu.edu.cn; 2 Department of Labor and Social Security, Ginling College, Nanjing Normal University, Nanjing 210097, China

**Keywords:** child gender, rural–urban migrant women, overwork, son preferences

## Abstract

Overwork is one of the risk factors for the work-related burden of disease. In China, nearly a quarter of migrant women are overworked. Working long hours can significantly increase the possibility of migrant women suffering from hypertension and hyperglycemia. The phenomenon of overtime work of migrant women and their health conditions deserves attention. Based on the China Migrants Dynamic Survey in 2016, this study indicates that giving birth to a boy may exacerbate overtime work among migrant women and having more boys in a family increases the probability of women’s overwork. Empirical results confirmed the existence of son preferences in China. Compared with women who gave birth to boys, women who gave birth to girls have a lower probability of being a workaholic due to a future fertility plan. Furthermore, the overwork of women is also due to the great economic burden on families to buy a wedding house, brought on by the birth of boys. To overcome the endogenous problem caused by this omitted explanatory variable, this study uses each province’s relative sex ratio at birth in 2010 as the instrumental variable of the firstborn’s gender. The IV results illustrate that the birth of boys still significantly exacerbates women’s overwork. Furthermore, considering age heterogeneity, the influence of son preference on women’s overtime work exists throughout their labor life cycle. This paper provides a new perspective for understanding migrant women’s overtime work and their health issues in urban China.

## 1. Introduction

### 1.1. Background

Overtime work has drawn widespread attention all over the world [1,2,3,4]. Studies show that every third person in Japan, every fourth person in Ireland and Austria, and every fifth person in the UK and Germany works longer than 48 h per week [5]. Overtime work is also common in the USA. About 11% of women and 26% of men in America worked more than 50 h per week [6]. Long working hours can have a wide range of negative consequences through its harmful effects to worsen workers’ lifestyle, such as circulatory system diseases [7,8], hypertension, diabetes, and the risk of suicide [9,10]. According to the report released by the WHO and the ILO in 2021, 488 million people from 194 countries have long working hours, and more than 745,000 people have died from heart disease and stroke related to working more than 55 h per week. Moreover, many studies indicate that overtime working can deteriorate workers’ mental health [4,11,12,13,14,15,16]. Hitherto, overwork has been identified as the greatest risk factor for the work-related burden of disease [17].

With the rapid development of the economy, the phenomenon of 996 overtime (working 6 days a week from 9 am to 9 pm) is particularly common in China, and there are news reports of “KAROSHI” (death by overwork) every year [18]. Moreover, the impact of overtime work on health differs significantly by gender, with women being more susceptible to the negative psychological effects caused by excessive labor hours [19]. Women’s overtime working increases their health risks due to worksite hazard exposure [20]. Among women, long work hours are also associated with poor mental health status and hypertension [21]. Worsened psychological distress resulting from longer working hours is widely documented in the literature, and women are at greater risk of psychological distress than men [22].

Despite all the negative effects of overtime working on workers’ physical and mental health, Chinese migrant women continue to work long hours with a high work intensity, whereas migrants work fewer hours than natives in the western and southern European countries studied [3]. According to the 2019 “Green Paper on Mental Health of Women in the Chinese Urban Workplace”, about 85% of working women have experienced symptoms of anxiety or depression, and the primary factor that triggers women’s physiological health and mental health problems is excessive workload and work pressure. Overwork is more severe among migrant women who migrate from rural areas to cities than among urban women. Migrant women face higher work intensity, with an average weekly working time of 55.06 h, compared with 37 h for urban women; thus, it is critical to investigate the motivation behind their overwork [23].

### 1.2. Literature Review

A significant volume of research has been conducted on the influencing factors of overwork in developed countries, such as Japan and the Netherlands, concerning individual characteristics, corporate culture, and social structural factors [24,25,26]. According to some studies, due to the temporary nature of migration, migrants who must return to poorer villages have greater incentives to work long hours than those who must return to more affluent villages [14]. For women, their work burden has its own peculiarities and is highly vulnerable to familial and maternity factors [1,27,28]. Although China issued the *Beijing Platform for Action* as early as 1995, and implemented systematic policies on gender equality and women’s empowerment, there is still a severe problem of gender inequality and son preference in China [29,30,31]. According to the Global Gender Gap Report (2020), China ranks last in the indicator of women’s “health and survival”. The score of health and survival was 0.926 in 2020, ranking 153rd in the world, which was worse than the ranking of 113th in 2006, with a score of 0.936. This means that China’s preference for boys is still severe.

The birth of boys has significant negative effects on women. Research shows that a woman’s risk of death increases by 7% per year for each son born, indicating that the gender of family children is closely related to their mother’s health [13]. The birth of a boy in can also aggravate the family’s economic pressure, resulting in the “overwork-family conflict” faced by married women, which is much more serious than that faced by men [29,32,33]. In addition, when compared with families without sons, families with sons have larger housing construction areas and are more likely to own multiple houses [34]. Therefore, having sons with no real estate will prompt married women in the family to work harder, and thus jointly undertake family economic responsibilities [35]. Although the literature has studied the impact of the birth of a boy on married women’s health and economic stress, there is a lack of research on the overwork burden of migrant women.

**Figure 1:**
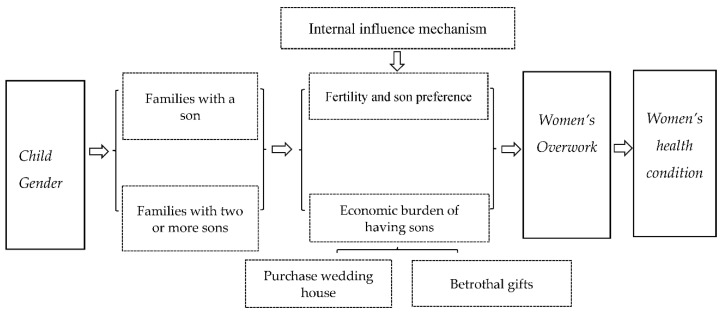
The theoretical framework of child gender and migrant women’s overwork in China.

Based on the abovementioned literature review, the realistic background of the high sex birth rate, and the poor health conditions for women in China, this paper summarizes the theoretical framework of son preference and married migrant women’s overtime work (see Figure 1). The internal influence mechanism concerns how the birth of boys promotes migrant women’s overtime by verifying the fertility preference and income spillover effect.

The main contributions of this paper are threefold. First, this paper is the first to study the overwork and health problems of married migrant women in China from the perspective of child gender. Second, this study analyzes the internal influence mechanism on how the birth of boys promotes migrant women’s overwork by verifying the gender preference for children and the elasticity of the employment-wage. The study of migrant women’s overwork can help promote the design of labor protection policies for migrants and improve the health level of female workers. Third, this paper also discusses the endogenous problems associated with overwork with the instrumental variables, as well as women’s overwork from the perspective of the life cycle, which increases the reliability and robustness of the conclusion.

The rest of this paper is arranged as follows: Section 2 reviews the data and methodology. Section 3 presents the summary of the variables model and the analysis of the empirical results. Section 4 discusses the internal mechanism of the child gender effect on migrant women’s labor participation. Section 5 presents the discussion and the conclusion.

## 2. Data and Methodology

### 2.1. Study Sample

This study used Volume A of the China Migrants Dynamic Survey (CMDS), which are national first-hand data published by the Migrant Population Service Center of the National Health Commission in 2016. It has been eight years since the data were first collected in 2009. This survey aimed to investigate the migrants who have currently resided in their inflow city for more than one month but have not registered their residence (*Hukou*) in the district. The survey was nationally representative and covered 31 provinces in China. The samples were obtained using the probability proportionate to size sampling (PPS) method with hierarchy. The questionnaire involved migrants’ demography characteristics, weekly working hours, and children’s primary caregivers, which provided a national sample of migrant women’s overwork for this study.

Based on the data questionnaire, the study selected the sample of migrant married women of 20–59 years old. The chosen age bracket is based on the Marriage Law of the People’s Republic of China that the legal age for marriage is 20 years for females and 22 years for males. Further, the average age for marriage in Chinese rural areas is lower than the national legal age for marriage, with the youngest married migrant women in this paper set at 20 years old. After sample selection, the samples of 42,158 migrant women were collected, including 11,290 one-girl families, 16,408 one-boy families, and 14,460 two-child families (including 2496 two-girl families, 3113 two-boy families, 3288 families with a firstborn as boy then girl, and 5563 families with a firstborn girl then boy).

### 2.2. Concept Definition and Variable Selection

(1) Migrant women. Migrant women mainly refer to rural–urban migrants. Based on the questionnaire, married migrant women are those: (1) with their marital status as married; (2) aged 20–59; (3) moved from rural areas to urban areas; (4) with children under the age of 18.
(2) Overwork. This is the dependent variable used to reflect the greatest risk factor of work-related disease burden, defined by long working hours. Referring to the health burden report released by the WHO and the ILO, people who work for more than 55 h have an increased risk of stroke and death due to ischemic heart disease, compared with those who work 35–40 h a week [17]. This paper defined overwork by using the weekly working time of no less than 55 h. The working hour was measured by two survey questions: “Have you done paid work for more than one hour before May Day?” and “How many hours have you worked this week?” Therefore, the migrant women can be divided into two categories: overtime workers (no less than 55 working hours) and nonovertime workers (less than 55 working hours).
(3) Child gender. This is the independent variable, indicated by the child’s gender in the family. Based on their family size (number of children), migrant women are divided into two categories: one-child family and two-child family. For the one-child family, there are one-boy families and one-girl families (basic group), while the two-child family is divided into four groups based on the child’s gender and birth order: two-girl families (basic group), with the firstborn girl then boy family, the firstborn boy then girl family, and the two-boy family.
(4) Childcare. This is one of the control variables, measured by the survey question: “Who is the primary caregiver of the child?” According to the original questionnaire, we defined the groups who are mainly taken care of by their mothers as 1, while those who answered that the main caregivers were fathers, grandparents, other relatives, neighbors and friends, teachers’ trusteeship, and unattended were assigned 0.
(5) Medical insurance. This control variable pertained to the participation of employees’ medical insurance, which helps to describe the health security level of migrant women. In the questionnaire, this variable is measured by the question: “Do you participate in the medical insurance for urban employees?” We defined the samples who participated in medical insurance as 1, while those who did not participate the employee’s medical insurance as 0.
(6) Labor contract. In the questionnaire, the nature of migrant women’s labor contract is divided into three types: (a) fixed term contract; (b) nonfixed term contract; and (c) no contract. In China, workers who sign the first two types of contracts are generally engaged in formal work, while those who do not sign contracts are generally engaged in informal work. Therefore, we defined the first two categories as 1 and those who did not sign a labor contract as 0.

### 2.3. Analytic Strategy

As explained by the variables above, the key to defining the overwork is to determine whether migrant women worked for more than 55 h a week. For each migrant woman, the value of overwork is either 1 or 0. As the overwork choice is a 0, 1 variable, this study used the binary Probit model to analyze the possibility of migrant women being overworked empirically. The specific model is as follows:

(1) $$pr(y_{i}=1|x_{i})=E(y_{i}|X)=\frac{1}{\sqrt{2\Pi }}\int_{-\infty }^{x_{i}\beta }e^{-\frac{t^{2}}{2}}dt$$

Assuming that there is a potential variable $y_{i}^{*}$, the actual observed value is $y_{i}$.

(2) $$y_{i}=\{\begin{array}{c} 1,y_{i}^{*}>0\\ 0,y_{i}^{*}\leq 0 \end{array};\left(Y_{i}^{*}=\alpha +\beta_{i1}X+\beta_{i2}X_{i}+\varepsilon_{i}\right)$$

The dependent variable $y_{i}$ in the model is dichotomously coded as 1 if migrant women worked for more than 55 h a week, and 0 if the weekly working hours of migrant women were less than 55 h. In addition, this paper includes those who had a job but were not working that week due to pregnancy, lactation, illness, or job training. The independent variable *X* in Equation (2) is the child’s gender. The control variables include women’s age, age square, education level, spouse’s education level, monthly family expenditure, monthly family income, childcare, medical insurance, labor contract, and migration pattern.

## 3. Results

### 3.1. Summary of Variables

Based on the descriptive statistics of the variables, the weekly working time of Chinese migrant women is revealed to be 54.41 h. Nearly a quarter of migrant women are overworked. That is, 39% of them work more than 55 h a week, and 22.2% of them work more than 60 h a week. Under such high-intensity work pressure, the medical security of migrant women is very insufficient. Only 13% of them have participated in urban employees’ medical insurance. This may be related to the nature of the employment of migrant women. According to the statistics on the nature of labor contracts, nearly 38% of migrant women have not signed any labor contract, let alone any labor protection. Besides, women are the main caretakers of children in the family. Whether for the first child or the second, the proportion of children mainly taken care of by their mothers is higher than 70%. The average age of migrant women is 35, and most of them only have junior or senior high school education. In the week prior to the survey, 72% of the participants had worked for more than one hour (including self-employed activities, as employers, or other forms of labor remuneration). The average number of children in a family is 1.4. The average monthly income of a local working family is RMB 6796, and the average monthly expenditure is RMB 3493. In general, for families with children, the probability of the firstborn being a boy is slightly higher than that of the firstborn being a girl, therefore aligning with the natural law of the childbirth gender. However, whether the first child is a girl or a boy, the probability of having a second-born boy is significantly higher than that of having a second-born girl. This may explain that there is a preference for the selective birth of boys among Chinese migrant women. The specific variable characteristics are shown in Table 1.

**Figure 2:**
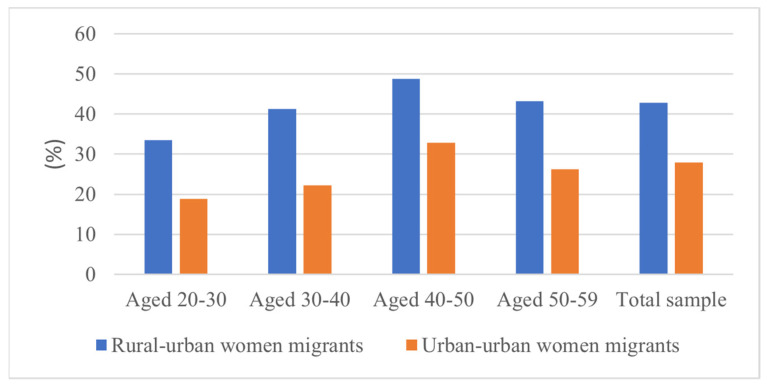
The weight of overwork between rural–urban and urban–urban women migrants in China.

Further, we analyzed the overwork of migrant women in different age groups from the perspective of the life cycle. The results in Figure 2 reveal that for rural–urban migrant women, the phenomenon of overwork is evident during their whole life cycle. The proportion of overwork increased in an inverted U-shape with age, with the possibility of overwork being greatest between the ages of 40 and 50. Based on the outflow area of migrant women, we also selected another sample of urban–urban migrants (moving from an urban area to another urban area) for comparative analysis. The results in Figure 1 indicate that at any age, the overwork of rural–urban migrant women is more severe than that of urban–urban migrant women. Therefore, in the following empirical analysis, we focused mainly on the rural–urban migrant women.

### 3.2. Birhthing Sons and Overwork of Migrant Women

In this part, we analyzed the causes of migrant women’s overwork from the perspective of child gender in the family. The results of Model 1 listed in Table 2 indicate that in only child families, the probability of being overworked for women who give birth to a boy is significantly higher, by 8.9%, than that of women who give birth to a girl. When analyzing the women’s labor supply in the labor market, the family economic level should be controlled for. Therefore, in Model 2, the control variables pertaining to the average monthly income of the family and the average monthly consumption expenditure are added. The results indicate that the influence of the firstborn boy on women’s overwork is also significantly positive. After further controlling for the childcare factors and migration pattern, the results are still significant. The impact of child gender on migrant women’s overwork is also reflected in two-child families. The empirical results of Model 4 indicate that, if the first child is a girl, a woman who has a boy as the second child is significantly more likely to be overworked than a woman who has a girl as the second child. Meanwhile, the overworking hours of women with two boys are significantly higher than that for women with two girls. As a result, even in two-child families, the birth of a boy increases the likelihood of women being overworked in the labor market. Furthermore, the nature of labor contracts is linked to women’s overwork. Compared with the migrant women in informal employment without contracts, migrant women who sign fixed-term contracts and nonfixed term contracts are significantly less likely to overwork. The results of Table 2 also show that participation in employees’ medical insurance helps to alleviate the pressure of migrant women’s overwork.

### 3.3. Number of Sons and the Burden of Migrant Women’s Overwork

The number of boys in a family was considered as an explanatory variable to analyze the overtime work of migrant women in different family structures. Based on the number of boys, families are divided into no-boy families (one-girl families and two-girl families), one-boy families, and two-boy families. The empirical results of Models 5–7 in Table 3 indicate that the number of boys in a family has a significant role in promoting migrant women’s overwork, and the results are highly significant when using Probit, Logit, and OLS for comparative analysis.

### 3.4. The Negative Effect of Overwork on Migrant Women’s Health Condition

Based on the above statistical analysis, migrant women not only bear the main burden of taking main care of children in the family, but the phenomenon of overworking in the workplace is also severe. These are not conducive to women’s physical and mental health, which is also the primary starting point of this paper studying the overwork of Chinese migrant women. As there were no relevant health variables in the 2016 CMDS dataset, we could not verify the indirect impact of having boys on the health of migrant women. In the questionnaire of the latest dataset in 2017, the family variables of child gender are missing, but there is information on migrant women’s overwork and health conditions. Using the binary Logit model, we examined the physical illnesses of migrant women in the past year. The results of Model 8 in Table 4 show that overwork can significantly increase the probability of physical illness for migrant women. Further, we analyzed the specific illness of hypertension and hyperglycemia in migrant women. The empirical results of Model 9 and Model 10 indicate that overwork can significantly increase the possibility of women suffering from hypertension and hyperglycemia. In a word, overwork plays a negative role in promoting the health status of migrant women.

## 4. Discussion

### 4.1. The Internal Mechanism of Child Gender and Women’s Overtime Work

From the above analysis, child gender has a significant impact on the overtime work of migrant women. Why does giving birth to boys improve the probability of being overworked for migrant women? According to the existing literature, migrant women continue to have son preferences [21] and giving birth to boys increases the likelihood of being overworked for migrant women. Women who give birth to a girl as the firstborn face more demand for a boy in the future, and they may reduce the probability of overwork to prepare for the birth of boys. By contrast, the birth of a boy imposes the expected economic burden on the family. Women are more likely to work long hours in the labor market because they have to share economic responsibility. Therefore, the influence of child gender on migrant women’s overwork can be explained from two perspectives: son preference effect and family expected income spillover effect.

#### 4.1.1. Son Preference and Migrant Women’s Fertility Behavior

Based on the principle of the “son-stop rule”, families with girls are willing to continue having more children until boys are born and families with a girl as the firstborn are willing to have more children until they have boys [36]. Women who have girls also have shorter breastfeeding periods so that they can prepare for the subsequent pregnancy [37]. For families with a boy as the firstborn, their desired children number is smaller than for those with girls [30]. This shows that son preference continues to exist. Such a child gender preference can affect women’s employment indirectly by influencing their fertility behavior. In the questionnaire, for women who are not working, there is related question on “being unemployed due to pregnancy or childbirth.” Based on this question, it is helpful to verify whether women with a girl as the firstborn are more likely to continue bearing children and being less likely to overwork. The main items related to the son preference effect are listed in Table 5. The empirical results of Models 11–13 indicate that with migrant women with firstborn girls as the basic group, those with firstborn boys have a lower probability of overwork due to pregnancy. In families with only daughters, or with unideal gender structures, the contraceptive use rate is lower and the rate of taking long-term contraceptives and sterilization measures is also lower, leading to the interruption of women’s labor participation for a period after giving birth to girls, as they choose to reduce their working hours and prepare to give birth to boys [38]. That is, women who have girls are more likely to continue bearing children and reduce their overwork time in advance in the labor market.

#### 4.1.2. The Economic Burden of Having Boys and Migrant Women’s Overwork

For migrant families in China, having boys means that they may have to face high marriage costs in the future, such as building their own houses or purchasing commercial houses, and preparing expensive betrothal gifts in advance for the bride’s family. Therefore, families with boys are more likely to buy houses in advance. With the development of China’s commercial housing market in the 1990s, housing prices have constantly been rising, and the purchase of real estate brings greater economic pressure on families with boys. Moreover, the gender imbalance has further exacerbated housing price growth [36]. Hence, women, as family members, gradually participate in the job market and share the economic responsibilities of the family. Therefore, the promotion effect of having boys on their mothers’ labor participation is attributed to the economic pressure caused by the need to purchase a wedding house. In Table 6, for the families with firstborn girls, buying a house has no significant effect on women’s overwork. However, for families with firstborn boys, the probability of being overworked is significantly higher for women without houses than for those with houses. This is consistent with the existing research conclusions; for example, Li and Wu (2016) proposed that for families with sons but no real estate, parents are more likely to earn money in advance to buy a house [22].

### 4.2. Endogeneity and Robustness Test

To verify the robustness of the previous estimation results, this study further considers that the model may have estimation deviation due to endogeneity. Owing to the spatial mobility of migrants, this study cannot precisely track the migrant women from the questionnaire. The selected groups might not have included the migrant women who had a daughter but returned to their hometown to give birth to a boy, or those who were not selected because of being overworked at their workplace. Hence, the empirical results might deviate. Therefore, the above results are further examined for endogeneity.

#### 4.2.1. Endogenous Problems

To overcome the endogeneity caused by omitted variables, this study uses an instrumental variable method for two-stage estimation. Based on data from the 2010 national census, the average sex ratio at birth in China is 116.86. Provinces with a sex ratio at birth higher than the national average level are defined as the areas with strong son preference (assigned as 1), and the provinces with a sex ratio at birth lower than the national average level are defined as the areas with weak son preference (assigned as 0). This binary dummy variable is used as the instrumental variable of the child’s gender. First, we tested the validity of the instrumental variables. The Wald test of Model 18 in Table 7 shows that the estimation of the instrumental variable’s coefficient is significant at the 1% level, which supports the exogenous hypothesis of the explanatory variables. Subsequently, to estimate the IV Probit model, we used the two-stage estimation method. In the first stage estimation of the IV Probit regression model, the estimation coefficient of the instrumental variable is significant at the 5% level. The estimated F statistic result is 12.13, which is much larger than 10 or the critical value of the weak instrumental variable test [39]. Therefore, we believe that the instrumental variables are highly correlated with the endogenous explanatory variables. The results in the second stage indicate that, when endogenous bias was controlled for, having a boy as the firstborn has a significant effect on the probability of being overworked in migrant women at the 10% level.

#### 4.2.2. Having Sons and Overwork from the Perspective of Women’s Life Cycle

As women’s reproductive behavior is closely related to age, there is heterogeneity in migrant women’s labor decision-making behavior across age groups. According to the statistics in Table 1, the average age of migrant women is 35 years, and 45 years is often defined as the end of the childbearing age. Therefore, based on these two important time nodes of 35 and 45 years, the migrant women are divided into three subsamples: 20–35 years old, 35–44 years old, and 45–59 years old. The specific empirical results are shown in Table 8. The results of Models 20–21 indicate that having boys has a positive effect on overtime working of women throughout their work life cycle. Moreover, the impact factor of the 45–59 age group is more evident than that of the 35–44 age group. This can be explained by the fact that at the initial stage of the working age, women aged 35–44 are more likely to have school-age children, and the economic pressure from the need to purchase houses for marriage is still prevalent. However, for women aged 45–59, families are facing increasing economic pressure from their sons to get married and buy marriage houses; therefore, women have a greater incentive to earn money through overwork.

## 5. Conclusions

Anthropologists’ surveys show that, since ancient times, women have invested more in their offspring’s childcare than men [40]. Our study found that migrant women in China are not only the primary caregivers of children in the family, but also their overtime work in the labor market is apparent. Moreover, overwork has a highly adverse impact on women’s health, which deserves social attention. Based on the CMDS data in 2016, this study empirically analyzes the cause of migrant women’s overwork from the perspective of child gender, as well as the internal influence mechanism. The main conclusions are as follows: First, in only-child families, the probability of being overworked for women who give birth to a boy is significantly higher than that of those who give birth to a girl. Even in two-child families, the birth of a boy can significantly prompt the probability of women overworking in the labor market. Second, the internal influence mechanism can be explained by the son preference effect and the expected the income spillover effect. Meanwhile, women who have a girl as their firstborn face an increased demand for a boy in the future, and they may be less likely to overwork to prepare for the birth of boys. By contrast, families with boys have to face economic burdens in the future. For families with firstborn boys, the probability of being overworked is significantly higher for women without houses than for those with houses. Third, having boys can aggravate migrant women’s overwork throughout their whole work life cycle, and the effect is especially significant for women after the age of 35.

This study has some implications for China’s social policy. When compared with rural women who used to take on the mother role as the primary caregiver, migrant women face more pressure throughout their lives. Migrant women require more attention from social communities and policy support, such as improving the system of childcare services, providing stable housing for the migrants, improving the living environment, and alleviating the economic pressure caused by the purchase of the real estate. At the same time, social groups should also pay attention to the health problems of migrant women caused by overtime work. Through the intervention of the government or a third party, the corresponding psychological consultation programs should be provided to improve their physical and mental health. Meanwhile, the medical insurance system for migrant women needs to be improved. In China, only 13% of migrant women participated in the employee medical insurance in the inflow area. Once they get sick, they will face the possibility of returning to their rural hometown or delaying treatment, which is not conducive to their health condition. The medical insurance system for migrant women in cities needs to be improved. The local government can organize regular physical examinations of migrant women, speed up the realization of off-site medical treatment, and gradually improve the medical security and welfare system of migrant women. Most importantly, the public has a long way to go in advocating gender equality between boys and girls since childbirth. Despite the United Nations agreeing on the landmark United Nation System-wide Action Plan on Gender Equality and the Empowerment of Women in early 2012, children still face gender inequality since birth in some countries. Not only do women face gender inequality within the same generation, but our study proves that the child gender of the next generation will also have an adverse impact on their health by affecting their overwork.

As for its limitations, this study did not consider the influence of fathers’ working time, their participation in the care of children, and the impact on migrant women’s overwork. The questionnaire only involves the primary caregivers of children; hence, there is no specific reference index of care time. Therefore, empirical analysis needs to be deepened, and further systematic research should be carried out in this direction.

## Figures and Tables

**Table 1:** Descriptive statistics of variable characteristics.

Variables	Explanation	Average	SD	Min.	Max.	Number
Overwork	Worked for more than 55 h a week (1 = yes; 0 = no)	0.39	0.49	0	1	38,375
Working hours	How many hours have you worked this week?	54.41	18.58	0	99	38,375
Age of firstborn		12.24	8.61	0	18	42,158
Gender of firstborn	0 = female; 1 = male	0.54	0.50	0	1	42,158
Gender of second child	0 = female; 1 = male	0.58	0.49	0	1	14,460
Primary caregiver of first child	1 = mother; 0 = others	0.71	0.45	0	1	41,492
Primary caregiver of second child	1 = mother; 0 = others	0.72	0.44	0	1	14,460
Labor contract	Type of labor contract (1 = fixed term labor contract; 2 = nonfixed term labor contract; 3 = working without a contract)	1.77	0.68	1	3	14,265
Medical insurance	Involved in the medical insurance system for urban employees (1 = yes; 0 = no)	0.13	0.33	0	1	38,375
Age	Rural–urban migrant women’s age	35.39	8.61	20	59	42,158
Women’s education	1 = primary school and below; 2 = middle school; 3 = high school; 4 = undergraduate and above	2.15	0.84	1	4	42,158
Spouse’s education	1 = primary school and below; 2 = middle school; 3 = high school; 4 = undergraduate and above	2.28	0.82	1	4	41,255
Monthly income	Self-income of last month (yuan)	3395.24	2769.18	−5000	90,000	34,422
Family income	Family income of last month in the local area (yuan)	6795.88	5178.54	0	200,000	42,158
Family expenditure	Family expenditure of last month in the local area (yuan)	3493.83	2368.03	200	50,000	42,158

**Table 2:** Having sons and migrant women’s overtime work.

Variables	Model 1	Model 2	Model 3	Model 4
	Marginal Effect	Marginal Effect	Marginal Effect	Marginal Effect
Firstborn boy (basic group: firstborn girl)	0.072 ***	0.068 ***	0.051 **	
	(4.99)	(4.22)	1.95	
Firstborn girl then boy (basic group: firstborn girl then a girl)				0.079 *
				(1.62)
Firstborn boy, then girl				0.073 *
				(1.43)
Firstborn boy, then boy				0.112 **
				(2.57)
Age of female	0.090 ***	0.071 ***	0.067 **	−0.079 *
	(6.65)	(5.23)	(2.54)	(−1.82)
Square of female age	−0.001 ***	−0.001 ***	−0.001 ***	0.001
	(−5.49)	(−4.21)	(−2.34)	(1.60)
Education level of female				
Junior middle school (basic group: elementary and below)	−0.018	−0.065 *	−0.185 ***	−0.114 *
	(−0.50)	(−1.79)	(−2.76)	(−1.72)
High school	−0.087 **	−0.162 ***	−0.389 ***	−0.230 **
	(−2.13)	(−3.89)	(−5.13)	(−2.47)
Undergraduate and above	−0.300 ***	−0.401 ***	−0.688 ***	−0.542 ***
	(−5.93)	(−7.80)	(−7.28)	(−3.26)
Education level of spouse				
Junior middle school (basic group: elementary and below)	0.044	0.016	−0.110	−0.080
	(1.28)	(1.38)	(−1.41)	(−1.04)
High school	−0.062	−0.118 ***	−0.230 ***	−0.302 ***
	(−1.52)	(−2.42)	(−2.71)	(−3.05)
Undergraduate and above	−0.386 ***	−0.489 ***	−0.324 ***	−0.258
	(−7.12)	(−9.12)	(−3.24)	(−1.61)
Monthly disposable income of family		0.212 ***	0.050 **	0.098 ***
		(18.89)	(2.02)	(2.86)
Monthly family consumption		0.118 ***	0.149 ***	0.095 *
		(5.67)	(3.99)	(1.78)
Labor contract (basic group: working without a labor contract)				−0.137 ***
				(−4.05)
Medical insurance (basic group: involved no employees’ medical insurance)				−0.336 ***
				(−5.64)
Primary caregiver of the first child (basic group: mother care)			0.170 ***	0.036
			(4.97)	(0.50)
Primary caregiver of the second child (basic group: mother care)				0.134 *
				(1.83)
Intraprovincial migration (basic group: Intra city migration)			−0.059	−0.117
			(−1.25)	(−1.50)
Interprovincial migration			−0.095 **	−0.025
			(−2.04)	(−0.35)
Intercept	−2.934 ***	−3.998 ***	2.343 ***	1.863 **
	(−7.68)	(−15.54)	(4.27)	(2.18)
Sample size	22,013	21,415	18,520	11,467

Note: ***, **, and * indicate significance at the levels of 1%, 5%, 10%, respectively.

**Table 3:** Empirical analysis of the number of boys and migrant women’s overwork.

Variables	Model 5	Model 6	Model 7
	Probit	Logit	OLS
One boy (basic group: no-boy)	0.068 **	0.110 **	0.026 **
	(2.04)	(2.04)	(2.03)
Two boys (basic group: no-boy)	0.085 **	0.137 **	0.032 **
	(2.16)	(2.15)	(2.15)
Other control variables	YES	YES	YES
Intercept	−3.670 ***	−5.944 ***	−0.867 ***
	(−8.44)	(−8.38)	(−5.33)
Sample size	11,467	11,467	11,467

Note: *** and ** indicate significance at the levels of 1% and 5%.

**Table 4:** Effects of overwork on migrant women’s health.

Variables	Model 8	Model 9	Model 10
	Poor Health	Hypertension	Diabetes
Overwork	0.076 ***	0.066 **	0.076 *
	(6.15)	(2.36)	(1.66)
Age of female	−0.022 ***	0.035 **	−0.048 **
	(−3.62)	(2.36)	(−2.34)
Square of female age	0.000 **	0.000 *	0.001 ***
	(2.27)	(1.66)	(3.95)
Education level of female			
Junior middle school (basic group: elementary and below)	−0.059 *	−0.169 ***	−0.119
	(−1.83)	(−3.54)	(−1.48)
High school and above	−0.038	−0.366 ***	−0.255 ***
	(−1.11)	(−6.28)	(−2.68)
The duration of the migration	0.008 ***	0.010 ***	0.014 ***
	(6.90)	(5.21)	(4.61)
lnwage	−0.025 ***	−0.046 ***	−0.045 ***
	(−4.57)	(−4.67)	(−3.08)
Medical insurance (basic group: involved no employees’ medical insurance)	−0.066 ***	0.018	−0.068
	(−5.04)	(0.62)	(−1.44)
Intercept	0.738 ***	−3.355 ***	−1.779 ***
	(6.19)	(−10.48)	(−4.20)
Sample size	40,898	39,980	39,980

Note: ***, **, and * indicate significance at the levels of 1%, 5%, 10%, respectively.

**Table 5:** Effect of a firstborn boy on the pregnancy of migrant women.

Variables	Model 11	Model 12	Model 13
	OLS	Probit	Logit
Firstborn boy (basic group: firstborn girl)	−0.029 ***	−0.178 ***	−0.338 ***
	(−4.01)	(−4.21)	(−4.25)
Primary caregiver of first child (basic group: mother care)	−0.011 ***	−0.076 ***	−0.162 ***
	(−8.79)	(−9.62)	(−10.26)
Other control variables	yes	yes	yes
Intercept	0.334 ***	−2.064 ***	−3.820 ***
	(3.16)	(−3.02)	(−2.91)
Sample size	7124	7124	7124

Note: *** indicate significance at the levels of 1%.

**Table 6:** Having sons, real estate purchases, and migrant women’s overtime work.

Variables	Model 16	Model 17
	Having Sons	Having Daughters
	Marginal effect	Marginal effect
Families without house (basic group: families with house)	0.137	0.106 **
	(1.55)	(2.12)
Labor contract (basic group: working without a labor contract)	−0.083	−0.223 ***
	(−1.28)	(−6.13)
Medical insurance (basic group: involved no employees’ medical insurance)	−0.593 ***	−0.624 ***
	(−5.39)	(−9.84)
Other control variables	yes	yes
intercept	5.685 ***	2.950 ***
	(3.89)	(3.33)
Sample size	12,050	14,499

Note: *** and ** indicate significance at the levels of 1% and 5%.

**Table 7:** Endogenous analysis of the influence of having sons on migrant women’s overwork.

Variables	Model 6	Model 18 IVProbit
	Probit	2SLS
First stage regression		
Provincial son preference in 2010		0.013 **
		(2.03)
Second stage regression		
Firstborn boy (basic group: firstborn girl)	0.051 **	4.000 *
	1.95	(1.73)
Other control variables	YES	YES
Intercept	2.343 ***	−6.069 ***
	(4.27)	(−5.91)
Wald’s test (*p*-value)	-	9.75 (0.002)
Sample size	18,520	26,550

Note: ***, **, and * indicate significance at the levels of 1%, 5%, 10%, respectively.

**Table 8:** Overwork of migrant women in different age groups.

	Model 19	Model 20	Model 21
	20–35 Years Old	35–44 Years Old	45–59 Years Old
Firstborn boy (basic group: firstborn girl)	0.054	0.136 ***	0.411 ***
	(1.98)	(3.58)	(2.79)
Other control variables	YES	YES	YES
Intercept	−4.591 ***	−2.210 ***	−3.318 **
	(−12.88)	(−10.95)	(−2.95)
Sample size	20,046	9339	603

Note: *** and **, indicate significance at the levels of 1% and 5%.

## Data Availability

The original data of this study were obtained from the Migrant Population Service Center, National Health Commission China. We are authorized to use the data by submitting a formal application to the Migrant Population service Center in November 2019, and the data are available online at the website http://www.chinaldrk.org.cn (accessed on 24 November 2021).
